# The β-adrenergic receptor-SGK1 signaling pathway in brown adipocytes protects GOT1 from proteasomal degradation

**DOI:** 10.3389/fcell.2025.1637770

**Published:** 2025-07-16

**Authors:** Chul-Hong Park, Minsung Park, J. Jason Collier, Ji Suk Chang

**Affiliations:** ^1^Laboratory of Gene Regulation and Metabolism, Pennington Biomedical Research Center, Baton Rouge, LA, United States; ^2^Islet Biology and Inflammation, Pennington Biomedical Research Center, Baton Rouge, LA, United States

**Keywords:** beta-adrenergic receptor, SGK1, GOT1, Brown adipose tissue (BAT), malate-aspartate shuttle (MAS), signaling/signaling pathways, ubiquitin-poteasome system

## Abstract

The malate-aspartate shuttle (MAS) is a key biochemical system that facilitates the transfer of reducing equivalents from the cytosol into mitochondria. It consists of two pairs of cytosolic and mitochondrial enzymes: glutamic-oxaloacetic transaminases (cGOT1, mGOT2) and malate dehydrogenases (cMDH1, mMDH2). We recently reported that cytosolic GOT1 is selectively elevated in brown adipocytes during cold exposure, while the expression of other MAS enzymes remains unchanged. Mechanistically, cold-induced activation of the β-adrenergic receptor (βAR)-cAMP-PKA signaling pathway promotes *Got1* transcription through the transcriptional coactivators PGC-1α and NT-PGC-1α. The resulting increase in GOT1 levels activates the MAS, thereby supporting mitochondrial respiration through enhanced fatty acid oxidation. In the present study, we identify the βAR-SGK1 (Serum- and Glucocorticoid-inducible Kinase 1) signaling axis as a novel regulatory mechanism that maintains GOT1 protein stability. SGK1 is activated downstream of βAR signaling in brown adipocytes during cold exposure. We show that expression of SGK1^S422D^, a constitutively active form of SGK1, protects GOT1 from ubiquitination by the E3 ubiquitin ligase RNF34 and subsequent degradation by the proteasome. Conversely, both pharmacological and genetic inhibition of SGK1 during βAR stimulation leads to a reduction in GOT1 protein levels without altering its mRNA expression. Together, these findings uncover a previously unrecognized role for the βAR-SGK1 signaling pathway in maintaining GOT1 protein stability in brown adipocytes, highlighting a multilayered signaling network that orchestrates metabolic adaptation during cold-induced activation.

## Introduction

Brown adipocytes are specialized fat cells that dissipate energy in the form of heat via mitochondrial uncoupling protein 1 (UCP1) (Cannon and Nedergaard, 2004; Nedergaard et al., 2001; Golozoubova et al., 2001). Notably, both cold exposure and pharmacological activation of brown adipocytes in adult humans have been associated with increased energy expenditure and improved insulin sensitivity (van Marken et al., 2009; Chondronikola et al., 2014; Ouellet et al., 2012; Hanssen et al., 2015; Cypess et al., 2015; O'Mara et al., 2020; Cypess et al., 2009; Saito et al., 2009), thereby positioning brown adipocytes as an appealing target for managing obesity-related metabolic disorders.

Cold exposure activates the sympathetic nervous system, leading to the release of norepinephrine, which stimulates β-adrenergic receptors (βAR) on brown adipocytes. This triggers the downstream cAMP-PKA signaling cascade that activates transcriptional coactivators, PGC-1α and its isoform NT-PGC-1α, which then promote the transcription of genes involved in mitochondrial thermogenesis and oxidative metabolism (Cannon and Nedergaard, 2004; Puigserver et al., 1998; Zhang et al., 2009; Chang et al., 2018). We recently showed that the βAR-cAMP-PKA-PGC-1α/NT-PGC-1α pathway induces the expression of *Got1*, which encodes GOT1, a key metabolic enzymes in the malate-aspartate shuttle (MAS) (Park et al., 2024). MAS is a biochemical system consisting of two mitochondrial transporters (AGC, OGC) and two pairs of metabolic enzymes located in the cytosol and mitochondria: glutamic-oxaloacetic transaminases (cGOT1, mGOT2) and malate dehydrogenases (cMDH1, mMDH2) (Borst, 2020). MAS facilitates the transfer of reducing equivalents (especially, electrons from NADH) from the cytosol into the mitochondrial matrix, where they can be used for mitochondrial respiration (Borst, 2020). We showed that cold-dependent induction of GOT1 in brown adipocytes activates the MAS, thereby supporting mitochondrial respiration through enhanced fatty acid oxidation during cold exposure (Park et al., 2024).

In this study, we further discovered that the serum- and glucocorticoid-inducible kinase 1 (SGK1) is activated downstream of βAR signaling in brown adipocytes during cold exposure, where it functions to protect GOT1 from proteasomal degradation independently of PKA-mediated transcriptional control. Our findings reveal that brown adipocytes employ both PKA- and SGK1-mediated pathways to fine-tune GOT1 function, underscoring the importance of integrated transcriptional and post-translational regulation in adapting to cold-induced metabolic demands.

## Materials and methods

### Animal studies

C57BL/6 mice (Jackson Laboratory, #000664) were housed at room temperature under a 12-h light/12-h dark cycle and maintained on a standard chow diet (5,001, LabDiet, St. Louis, MO) with *ad libitum* feeding. 9-to-14-week-old C57BL/6 mice were randomly assigned to experimental groups and were singly housed at room temperature or 4°C for 0.2, 1, 2, 3, and 4 days. At the end of experiments, mice were euthanized to extract brown adipose tissue (BAT) by carbon dioxide asphyxiation followed by cervical dislocation that is in accordance with the established recommendations of the American Veterinary Medical Association (AVMA) Guidelines for the Euthanasia of Animals. All animal experimental procedures were approved by the Institutional Animal Care and Use Committee of the Pennington Biomedical Research Center, and animal study reporting adheres to the ARRIVE guidelines (Kilkenny et al., 2010).

### Cell culture and transfection

HEK293 cells (ATCC, #CRL-1573) were maintained in DMEM supplemented with 10% FBS and 1% Penicillin/Streptomycin (Invitrogen) and transfected using Lipofectamine 3,000 (Thermo Fisher Scientific, #L3000008) in accordance with manufacturer’s instructions with the following plasmids: pCMV-GOT1-myc (OriGene, #MR206497), HA-Ubiquitin (a gift from Dr. Beth Floyd), HA-RNF34 (Addgene, #119938), pcDNA3.1-SGK1^S422D^ (Pao et al., 2010), and pcDNA3.1-SGK2^S356D^ (Pao et al., 2010) (gifts from Dr. Alan C. Pao).

### Brown adipocyte differentiation

Brown preadipocytes (Uldry et al., 2006; Jun et al., 2014; Kim et al., 2018) were grown to confluence in DMEM medium supplemented with 20 nM insulin and 1 nM T3 (differentiation medium) and induced for differentiation by incubating in differentiation medium supplemented with 0.5 mM isobutylmethylxanthine (IBMX), 0.5 µM dexamethasone, and 0.125 mM indomethacin for 48 h, as previously described (Chang et al., 2010). Thereafter, the cells were maintained in differentiation medium until day 7. Fully differentiated brown adipocytes were treated with MG132 (Sigma, #474790), isoproterenol (Sigma, # I-2760), KU0063794 (Tocris, #3725), GSK690693 (Tocris, #4144), GSK650394 (Cayman Chemicals, #17001), or Go 6,983 (Tocris, #2285).

### Generation of Sgk1-deficient brown preadipocytes

The stromal vascular fraction (SVF) containing brown preadipocytes was isolated from interscapular brown adipose tissue (BAT) of 4-days-old Sgk1^fl/fl^ pups (Fejes-Toth et al., 2008) (a gift from Dr. Aniko Naray-Fejes-Toth) by collagenase digestion and immortalized by infection with SV40T antigen-expressing retrovirus as previously described (Zhang et al., 2009). LoxP/Cre-mediated deletion of Sgk1 was then induced by retrovirus expressing Cre recombinase (Addgene, #34568) (Wang et al., 2010) as we performed previously (Park et al., 2024).

### Mitochondrial respiration assay

Oxygen consumption rates (OCR) of brown adipocytes were measured as described previously (Jun et al., 2014). Briefly, brown adipocytes (10^6^ cells) were placed in a magnetically stirred respirometric chamber of the OROBOROS Oxygraph-2k (Oroboros Instruments, Innsbruck, Austria) (Jun et al., 2014). The OCR measurements were obtained at baseline and after injections of oligomycin (an ATP synthase inhibitor), FCCP (chemical uncoupler), and antimycin A (a complex III inhibitor). Mitochondrial respiration was determined by subtracting antimycin A-independent non-mitochondrial respiration from total respiration. The coupled respiration refers to the mitochondrial respiration that is sensitive to oligomycin, and leak respiration represents oligomycin-independent mitochondrial respiration.

### GOT activity assay

Enzyme activity of glutamic-oxaloacetic transaminase (GOT) was measured in BAT tissue homogenates by the GOT Activity Assay kits (Sigma) in accordance with manufacturer’s instructions.

### Immunoprecipitation and Western blot analysis

Cells were lysed in 20 mM HEPES, pH 7.0, 150 mM NaCl, 0.2% NP-40 supplemented with protease and phosphatase inhibitor cocktail (Roche). Lysates were precleared with protein A-agarose beads and immunoprecipitated with antibody-coated beads for 3 h at 4°C as described previously (Chang et al., 2010). After washing, the immunoprecipitated proteins were subjected to Western blot analysis. Antibodies used were as follows: GOT1 antibody (Pro Sci, #30–379), Myc (9E10) antibody (Cell Signaling, # 2,276), HA antibody (Abcam, #ab9110), NDRG1 (D6C2) antibody (Abcam, #ab 9,408), p-NDRG1 (Thr346) antibody (Abcam, #ab 5,482), RNF34 antibody (Novus, #NBP2-56413), SGK1 antibody (Millipore, # 07–315), SGK2 antibody (Cell Signaling, # 5,595), α-tubulin antibody (Abcam, #ab7291), and β-actin antibody (Sigma, #A5441).

### Quantitative real-time PCR analysis

Total RNA was isolated and converted into cDNA through reverse transcription as described previously (Chang et al., 2010; Chang et al., 2012). Gene expression analysis was performed using the Applied Biosystems 7,900 (Applied Biosystems) and iTaq Universal SYBR Green Supermix (Bio-Rad). Relative mRNA abundance of the genes of interest was determined using gene-specific primers after normalization to cyclophilin mRNA by the 2^−ΔΔCt^ method. The validated primer sequences were obtained from PrimerBank public resource (Wang and Seed, 2003).

### Statistical analysis

All graphs were created by using Prism 10 software (GraphPad Software, San Diego, CA, United States) and Student’s t-test was used to compare differences between the groups. Data are presented as mean ± SEM. Values of *P* < 0.05 were considered statistically significant.

## Results

### GOT1 protein levels are regulated by the ubiquitin-proteasome pathway

**FIGURE 1:**
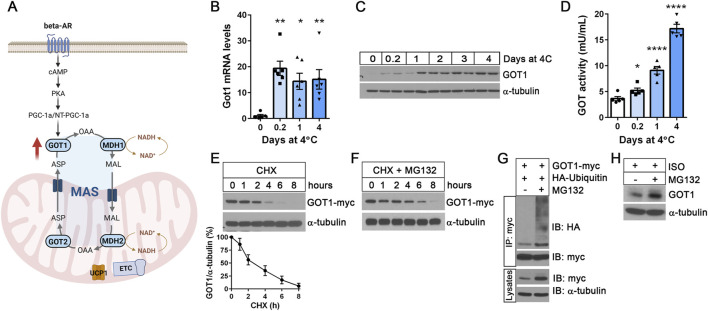
GOT1 protein levels are regulated by the ubiquitin-proteasome pathway **(A)** A schematic of the malate-aspartate shuttle (MAS) in brown adipocytes. MAS consists of two mitochondrial transporters and two pairs of cytosolic (GOT1 and MDH1) and mitochondrial (GOT2 and MDH2) enzymes. Cold-activated MAS facilitates the transfer of reducing equivalents (especially, electrons from NADH) from the cytosol into the mitochondria. The illustration was created using BioRender (https://www.biorender.com). **(B)** qPCR analysis of *Got1* gene expression in BAT during cold exposure. C57BL/6J female mice were housed at 23°C or exposed to 4°C for the times indicated. **(C)** GOT1 protein expression in cold-exposed BAT. **(D)** GOT enzymatic activity in BAT homogenates. **(E)** Degradation of GOT1 protein expressed in HEK293 cells upon treatment with 50 μg/mL of cycloheximide (CHX). A line graph shows the normalized GOT1 protein levels at the indicated time points from three independent experiments. **(F)** Co-treatment with MG132 attenuates proteasomal degradation of GOT1 in the presence of cycloheximide (CHX). **(G)** Ubiquitination of GOT1 protein immunoprecipitated from HEK293 cells treated with 10 µM of MG132 for 2 h. **(H)** Accumulation of GOT1 protein levels in brown adipocytes upon treatment with 10 µM of MG132 for 8 h. Brown adipocytes were stimulated with the β-adrenergic receptor agonist isoproterenol (ISO). All data are presented as the Mean ± SEM. *p < 0.05, **p < 0.01, ****p < 0.0001.

We recently reported that *Got1* gene expression is significantly upregulated by cold in brown adipocytes via the well-established βAR-cAMP-PKA-PGC-1α/NT-PGC-1α pathway, leading to the activation of the malate-aspartate shuttle (MAS) (Figure 1A) (Park et al., 2024). Intriguingly, a time-course analysis of *Got1* mRNA and protein levels in BAT during cold exposure revealed a temporal disconnect between their peak levels. *Got1* mRNA levels surged as early as 5 h of cold exposure and remained consistently elevated (Figure 1B), whereas GOT1 protein levels increased more gradually, reaching their peak between 1 and 2 days after the onset of cold exposure (Figure 1C). This trend in protein levels was closely paralleled the rise in GOT1 enzymatic activity during cold exposure (Figure 1D). Notably, GOT1 activity continued to increase at 4 days of cold exposure, despite no further increases in protein levels. These findings suggest that GOT1 protein expression, stability and activity may be regulated during cold exposure by multiple mechanisms, such as translational control, protein degradation, and post-translational modifications (Halbeisen et al., 2008).

Several proteomics studies discovered GOT1 as an ubiquitinated protein (Kim et al., 2011; Danielsen et al., 2011; Emanuele et al., 2011; Bartelt et al., 2018), with ubiquitination sites at K33, K276, K290 and K321 in murine BAT (Bartelt et al., 2018). Thus, we sought to examine whether GOT1 protein levels are regulated by the ubiquitin-proteasome pathway. First, we expressed GOT1-myc in HEK293 cells and assessed its stability after inhibition of protein synthesis with cycloheximide (CHX). GOT1 rapidly degraded following CHX treatment, showing that GOT1 is a short-lived protein with an estimated half-life of approximately 2.5 h (Figure 1E). Simultaneous inhibition of proteasome activity using the proteasome inhibitor MG132 significantly slowed down the degradation of GOT1 in the presence of CHX (Figure 1F), indicating that GOT1 is degraded via the ubiquitin-proteasome system. Indeed, MG132 treatment led to the accumulation of ubiquitinated GOT1 and disrupted protein turnover (Figure 1G). Next, we assessed the effect of MG132-mediated proteasome inhibition on GOT1 protein levels in brown adipocytes during βAR stimulation with the β-AR agonist isoproterenol (ISO). Similarly, MG132 treatment resulted in a modest increase in GOT1 protein levels in ISO-stimulated brown adipocytes (Figure 1H). Together, these results indicate that GOT1 is a short-lived protein, and its levels in brown adipocytes are regulated by the ubiquitin-proteasome pathway.

### RNF34 is an E3 ubiquitin ligase for GOT1

**FIGURE 2:**
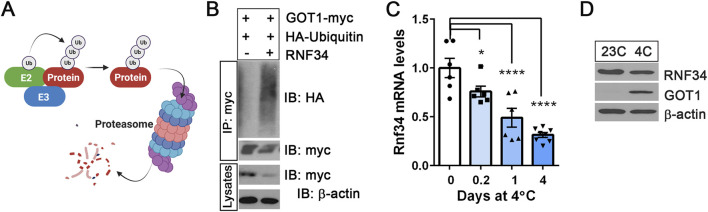
RNF34 is an E3 ubiquitin ligase for GOT1 **(A)** A schematic describing the ubiquitin-proteasome-dependent degradation of the protein. E2: E2 ubiquitin-conjugating enzyme, E3: E3 ubiquitin ligase. **(B)** RNF34-dependent ubiquitination and degradation of GOT1 in HEK293 cells. **(C)** *Rnf34* gene expression in BAT from mice exposed to 4°C for the times indicated. **(D)** RNF34 protein levels in BAT from mice housed at 23°C or 4°C for 7 days. All data are presented as the Mean ± SEM. *p < 0.05, **p < 0.01, ****p < 0.0001.

Ubiquitination involves the action of three enzymes: E1 ubiquitin-activating enzyme, E2 ubiquitin-conjugating enzyme, and E3 ubiquitin ligase (Hershko and Ciechanover, 1992). The E3 ubiquitin ligase interacts with both E2 and its target protein, promoting the transfer of ubiquitin from E2 to its target protein (Figure 2A). A previous proteomics study (Kristensen et al., 2012) identified GOT1 as an interacting protein of an E2 ubiquitin-conjugating enzyme UBE2H that can be complexed with an E3 ubiquitin ligase RNF34. Thus, we tested whether RNF34 functions as an E3 ubiquitin ligase for GOT1. Indeed, co-expression of RNF34 with GOT1 promoted GOT1 ubiquitination and its turnover (Figure 2B). Interestingly, RNF34 has been shown to be a cold-regulated E3 ubiquitin ligase in BAT (Wei et al., 2012). In line with previous findings that cold exposure downregulates *Rnf34* expression in BAT (Wei et al., 2012), we observed a time-dependent decrease in *Rnf34* expression in BAT during cold exposure (Figure 2C). However, this decrease had only a modest effect on RNF34 protein levels (Figure 2D). The previous study has shown that RNF34 specifically targets PGC-1α for ubiquitination, and its downregulation in BAT during cold exposure increases PGC-1α protein stability (Wei et al., 2012). In a similar manner, the observed reduction in RNF34 levels may affect the proteasomal degradation of GOT1 in cold-activated BAT. However, given the relatively modest decrease in RNF34 protein levels, it is likely that an additional mechanism contributes to inhibiting RNF34’s activity toward GOT1.

### The βAR-SGK1 signaling pathway increases GOT1 protein stability

Post-translational modifications, such as phosphorylation, have been shown to disrupt the interaction between E3 ubiquitin ligases and their target proteins (Hong et al., 2011; Agarwal et al., 2016; Liao et al., 2019). This interference affects the ubiquitination process, thereby altering protein stability and function. Thus, we sought to investigate whether the downstream signaling pathways of β-adrenergic receptors specifically influence GOT1 protein stability without altering its mRNA expression. In parallel to the βAR-cAMP-PKA signaling cascade, cold stress also activates the βAR-cAMP-Epac1-mTORC2 pathway in brown adipocytes, resulting in Akt activation via phosphorylation at serine 473 (Albert et al., 2016; Festuccia, 2025; Ye et al., 2019). Consistent with these earlier findings, we observed increased phosphorylation of Akt at Ser473 during cold exposure, with peak levels between 1 and 2 days (Supplementary Figure S1). Akt belongs to the AGC kinase family, which includes evolutionally related serine/threonine kinases such as protein kinase C (PKC) and serum- and glucocorticoid-induced kinase (SGK). mTORC2 is known phosphorylate both PKC and SGK (Garcia-Martinez and Alessi, 2008; Yan et al., 2008; Baffi et al., 2021); however, their activation status and functional roles in BAT under cold exposure remain to be elucidated.

**FIGURE 3:**
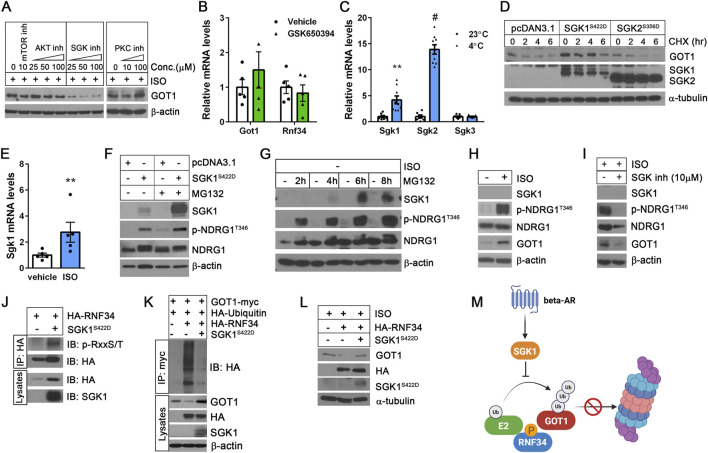
The βAR-SGK1 signaling pathway increases GOT1 protein stability in brown adipocytes **(A)** Effect of kinase inhibitors on GOT1 protein levels. Isoproterenol (ISO)-treated brown adipocytes were co-treated for 2 h with KU0063794, GSK690693, GSK650394, and Go 6,983 to inhibit mTORC2, Akt, SGK, and PKC, respectively. **(B)** No alteration of *Got1* gene expression by the SGK inhibitor GSK650394 (25 µM). **(C)** Effect of cold stress on the expression of *Sgk1*, *Sgk2*, and *Sgk3* isoforms in BAT. C57BL/6 male mice were housed at 23°C or exposed to 4°C. **(D)** SGK1 activation stabilizes GOT1. HEK293 cells co-expressing GOT1 and SGK1^S422D^ or SGK2^S356D^ were treated with 50 μg/mL of CHX. **(E)** *Sgk1* expression in brown adipocytes treated with vehicle or 10 µM of isoproterenol (ISO) for 4 h. **(F)** SGK1^S422D^-dependent phosphorylation of NDRG1 at Thr346 in HEK293 cells in the absence and presence of MG132. **(G)** Accumulation of SGK1 protein in brown adipocytes during treatment with 10 µM of MG132. **(H)** β-adrenergic stimulation of brown adipocytes with isoproterenol induces phosphorylation of NDRG1 at Thr346. **(I)** Pharmacological inhibition of SGK1 blunts NDRG1 phosphorylation in ISO-stimulated brown adipocytes. **(J)** SGK1^S422D^-dependent phosphorylation of RNF34 in HEK293 cells. **(K)** SGK1^S422D^ inhibits RNF34-dependent ubiquitination and degradation of GOT1 in HEK293 cells. **(L)** SGK1^S422D^ mitigates the inhibitory effect of RNF34 overexpression on GOT1 in brown adipocytes. **(M)** A schematic describing the role of SGK1 in enhancing GOT1 protein stability by protecting it from ubiquitin/proteasome-dependent degradation. All data are presented as the Mean ± SEM. **p < 0.01, #*p* < 0.0001.

To determine whether mTORC2, Akt, PKC, or SGK signaling pathway regulates GOT1 protein stability independently of PKA-mediated transcriptional control, we examined the effects of their respective inhibitors on GOT1 protein levels in brown adipocytes during βAR stimulation with isoproterenol (ISO). Inhibition of mTORC2 by KU0063794 resulted in a modest decrease in GOT1 protein levels (Figure 3A). Interestingly, while inhibiting Akt with GSK690693 and PKC with Go 6,983 did not affect GOT1 protein levels, SGK inhibition by GSK650394 led to a significant decrease in GOT1 protein levels (Figure 3A). Importantly, this decrease in GOT1 protein levels was not due to reduced *Got1* gene expression (Figure 3B), indicating that SGK inhibition during βAR stimulation does not impact PKA-dependent *Got1* transcription. In addition, *Rnf34* expression was not altered by SGK inhibition (Figure 3B). These results indicate that the βAR-SGK signaling pathway regulates GOT1 at the post-translational level in brown adipocytes.

The SGK family consists of three isoforms: SGK1, SGK2, and SGK3 (Lang and Cohen, 2001). Our gene expression analysis of BAT revealed that expressions of *Sgk1* and *Sgk2* are highly induced by cold, whereas *Sgk3* expression remains low and unchanged (Figure 3C). Thus, we investigated the role of SGK1 and SGK2 in regulating GOT1 protein by co-expressing constitutively active forms of SGK1 (SGK1^S422D^) and SGK2 (SGK2^S356D^) (Pao et al., 2010), along with GOT1-myc in HEK293 cells. Phosphorylation of SGK1 at Ser422 and SGK2 at Ser356 within their C-terminal hydrophobic motifs by mTORC2 promotes their full activation by facilitating subsequent phosphorylation at the activation loop by PDK1 (Garcia-Martinez and Alessi, 2008; Yan et al., 2008). Interestingly, the expression of SGK1^S422D^ led to increased GOT1 protein levels at baseline and prevented GOT1 degradation during CHX treatment (Figure 3D). In contrast, SGK2^S356D^ had no impact on GOT1 protein levels prior to and during CHX treatment (Figure 3D). Together, these results suggest that SGK1 activation specifically protects GOT1 from proteasomal degradation.

Consistent with cold-dependent upregulation of *Sgk1* in BAT (Figure 3C), *Sgk1* gene expression was also induced by βAR stimulation in brown adipocytes (Figure 3E). However, we were unable to determine the effect on SGK1 protein levels due to its short half-life, which is approximately 30 min (Arteaga et al., 2006; Brickley et al., 2002; Zhou and Snyder, 2005). NDRG1 is a well-known substrate of SGK1 (Murray et al., 2004). Thus, NDRG1 phosphorylation at Thr346 by SGK1 is widely used as a surrogate marker for SGK1 activation (Murray et al., 2004; Murakami et al., 2010; McCaig et al., 2011; Inglis et al., 2009). In our study, we found that the SGK1^S422D^ protein is rapidly targeted for proteasomal degradation, as evidenced by its accumulation in the presence of MG132 (Figure 3F). Despite its rapid turnover, SGK1^S422D^ efficiently phosphorylated NDRG1 at Thr346 (Figure 3F), indicating that SGK1 ^S422D^ retains functional activity despite its short half-life. In a similar manner, MG132 treatment of brown adipocytes resulted in a time-dependent accumulation of SGK1 protein, accompanied by a corresponding increase in NDRG1 phosphorylation at Thr346 (Figure 3G). Thus, to evaluate SGK1 activation during β-adrenergic stimulation of brown adipocytes, we assessed the phosphorylation of NDRG1 at Thr346. As expected, treatment with the βAR agonist isoproterenol significantly increased NDRG1 phosphorylation at Thr346 (Figure 3H), indicating SGK1 activation in response to βAR signaling. This activation was associated with increased GOT1 levels. Conversely, pharmacological inhibition of SGK1 by GSK650394 in ISO-stimulated brown adipocytes blunted NDRG1 phosphorylation at Thr346 and decreased GOT1 protein levels (Figure 3I), without affecting *Got1* mRNA expression (Figure 3B). Taken together, these findings suggest that the βAR-SGK1 signaling axis regulates GOT1 at the post-translational level in brown adipocytes.

### SGK1 activation inhibits RNF34-dependent ubiquitination and degradation of GOT1

SGK1 has been shown to phosphorylate an E3 ubiquitin ligase NEDD4-2 (Zhou and Snyder, 2005), preventing NEDD4-2 from binding to its target substrates, such as sodium channels and anion transporters (Debonneville et al., 2001; Snyder et al., 2002; Wang and You, 2017). This action leads to increased stabilization of these proteins. SGK1 is 54% homologous to Akt in its catalytic domain, with both the kinases sharing the same phosphorylation consensus motifs (RxRxxS/T and RxxS/T) (Zhou and Snyder, 2005; Murakami et al., 2010; Debonneville et al., 2001; Brunet et al., 2001). Protein sequencing analysis revealed that RNF34 and GOT1 contain RxxS/T motifs (^97^RRCS ^100^, ^98^RCST ^101^ and ^168^RSQT ^171^ in RNF34; ^283^RVLS ^286^ in GOT1). Thus, we sought to test the hypothesis that SGK1 may phosphorylate RNF34 or GOT1, potentially disrupting their interaction required for GOT1 ubiquitination. To examine whether SGK1^S422D^ directly phosphorylates RNF34 or GOT1, we probed the immunoprecipitated proteins using a phospho-RXXS/T motif-specific antibody. SGK1^S422D^ was found to phosphorylate RNF34 (Figure 3J), but not GOT1 (data not shown). Moreover, the co-expression of SGK1^S422D^ with RNF34 and GOT1 attenuated RNF34-mediated ubiquitination of GOT1 and its subsequent proteasomal degradation (Figure 3K), indicating that SGK1^S422D^-dependent phosphorylation of RNF34 may impair its ability to interact with GOT1. However, we were unable to confirm whether SGK1^S422D^ disrupts the RNF34-GOT1 interaction, likely due to the highly transient nature of the interactions within the SGK1^S422D^-RNF34-GOT1 complex (data not shown).

Consistent with the observed *in vitro* findings, co-expression of SGK1^S422D^ with RNF34 in ISO-stimulated brown adipocytes alleviated the suppressive effect of RNF34 overexpression on GOT1 protein levels (Figure 3L). Collectively, these results support a model in which SGK1 activation enhances GOT1 protein stability in brown adipocytes by inhibiting its degradation via the ubiquitin-proteasome pathway (Figure 3M).

### Loss of SGK1 in brown adipocytes reduces GOT1 protein levels without affecting its gene expression

**FIGURE 4:**
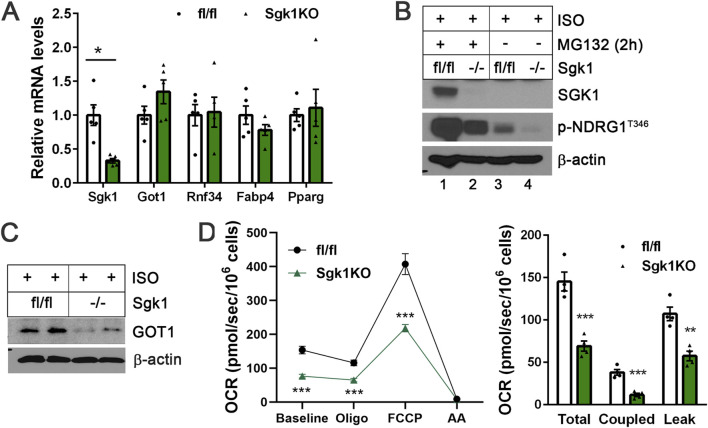
Loss of SGK1 reduces GOT1 protein levels in brown adipocytes without altering its gene expression **(A)** qPCR analysis of *Sgk1* ^fl/fl^ and *Sgk1* ^−/−^ brown adipocytes treated with isoproterenol (ISO) for 4 h. **(B)** Validation of SGK1 deficiency in *Sgk1* ^−/−^ brown adipocytes. **(C)** Loss of SGK1 reduces GOT1 protein levels in ISO-stimulated brown adipocytes. **(D)** Effects of *Sgk1* ablation on mitochondrial respiration in ISO-stimulated brown adipocytes. Left panel: The oxygen consumption rates (OCR) were measured in *Sgk1* ^fl/fl^ and *Sgk1* ^−/−^ brown adipocytes after 4 h of ISO stimulation, followed by injections of oligomycin (Oligo), FCCP, and complex III inhibitor antimycin A (AA). Right panel: Total mitochondrial respiration, oligomycin-dependent coupled respiration, and oligomycin-independent leak respiration were determined as described in the Methods section. All data are presented as the Mean ± SEM. *p < 0.05, **p < 0.01, ***p < 0.001.

To further validate the role of SGK1 in regulating GOT1 at the post-translational level, we generated *Sgk1*-deficient brown adipocytes. The stromal vascular fraction (SVF) cells were isolated from BAT of *Sgk1* ^fl/fl^ mice (Fejes-Toth et al., 2008), transduced with retrovirus expressing Cre recombinase to induce loxP/cre-mediated deletion of the *Sgk1* gene, and differentiated into brown adipocytes followed by treatment with isoproterenol for 4 h. *Sgk1* ablation did not affect brown adipogenesis, as shown by no difference in adipogenic gene expression (Fabp4, Pparg) between *Sgk1* ^fl/fl^ and *Sgk1* ^−/−^ brown adipocytes (Figure 4A). Loss of SGK1 was further confirmed by the blunted accumulation of SGK1 protein in the presence of MG132 (Figure 4B, lane 2). Moreover, the absence of SGK1 activity led to a significant reduction in NDRG1 phosphorylation at Thr346 in ISO-stimulated *Sgk1* ^−/−^ brown adipocytes (Figure 4B, lane 4). The residual NDRG1 phosphorylation may suggest the presence of an additional, albeit minor, kinase(s) capable of phosphorylating NDRG1 in the absence of SGK1 activity. Consistent with the effects of pharmacological SGK1 inhibition, the absence of SGK1 activity during β-adrenergic stimulation of *Sgk1* ^−/−^ brown adipocytes resulted in a decrease in GOT1 protein levels (Figure 4C), without altering *Got1* mRNA expression (Figure 4A). Additionally, *Rnf34* expression remained unchanged in *Sgk1* ^−/−^ brown adipocytes (Figure 4A). These results further support the conclusion that SGK1 signaling downstream of β-adrenergic receptors regulates GOT1 protein levels by protecting it from ubiquitin-proteasome-mediated degradation.

We recently showed that loss of GOT1 in brown adipocytes impairs ISO-stimulated mitochondrial respiration due to defective activation of the malate-aspartate shuttle (MAS) (Park et al., 2024). To investigate whether SGK1 activation influences mitochondrial respiration through enhancing GOT1 levels in brown adipocytes, we assessed mitochondrial respiration by measuring oxygen consumption rates (OCR) in ISO-stimulated *Sgk1* ^fl/fl^ and *Sgk1* ^−/−^ brown adipocytes. As expected, *Sgk1* ^−/−^ brown adipocytes exhibited reduced mitochondrial respiration compared to *Sgk1* ^fl/fl^ brown adipocytes, and their OCR remained lower following treatment with the ATPase inhibitor oligomycin and chemical uncoupler FCCP (Figure 4D, left panel). Both coupled respiration, which reflects ATP production via oxidative phosphorylation, and leak respiration, indicative of UCP1-mediated uncoupled respiration, were decreased in the absence of SGK1 (Figure 4D, right panel). These results suggest that reduced GOT1 levels in *Sgk1* ^−/−^ brown adipocytes may contribute to decreased mitochondrial respiration. However, it is also plausible that SGK1 regulates additional substrates that can influence mitochondrial respiration in brown adipocytes.

## Discussion

We recently reported that GOT1, one of the key enzymes in the malate-aspartate shuttle (MAS), is significantly upregulated in BAT during cold exposure, while other MAS enzymes like GOT2, MDH1, and MDH2 remain consistently expressed and do not show any significant changes (Park et al., 2024). This selective induction of GOT1 in BAT is driven by cold-dependent activation of the βAR-cAMP-PKA signaling pathway that promotes *Got1* transcription through PGC-1α and NT-PGC-1α. In this study, we further demonstrate that the βAR-SGK1 signaling pathway contributes to the elevation of GOT1 protein levels by protecting it from proteasomal degradation. Our *in vitro* findings indicate that SGK1 phosphorylates the E3 ubiquitin ligase RNF34, thereby inhibiting its ability to ubiquitinate GOT1 for proteasomal degradation. In support of SGK1′ role in the post-translational regulation of GOT1 protein stability, both pharmacological and genetic inhibition of SGK1 activity in brown adipocytes led to a significant reduction in GOT1 protein levels during βAR stimulation, without affecting its gene expression.

Cold stress has been shown to increase proteasome activity in BAT, accompanied by the upregulation of genes involved in the proteasome pathway (Chang et al., 2018; Bartelt et al., 2018). This activation is crucial for maintaining cellular protein quality and supporting the increased metabolic demands of thermogenesis. Our findings reveal that the βAR-SGK1 signaling pathway plays a protective role in maintaining GOT1 protein levels, thereby supporting the malate-aspartate shuttle, which is important for cold-induced metabolic adaptation in BAT. While our data highlights post-translational regulation of GOT1 via the ubiquitin-proteasome system, it remains unclear whether translational mechanisms also contribute to its regulation. Notably, microRNAs such as miR-2115-3p and miR-9-5p have been reported to bind to *Got1* mRNA and suppress its expression in trophoblast cells and pancreatic cancer cells, respectively (Deng et al., 2022; Wang et al., 2019). In addition, recent studies have shown that m^6^A methylation of mRNA can affect transcript stability and translation efficiency, influencing protein levels (Zhao et al., 2017; Zaccara et al., 2019; Xiao et al., 2024). Thus, we cannot rule out the possibility that GOT1 protein levels in BAT during cold exposure may be governed by a complex interplay of transcriptional, post-transcriptional, translational, and post-translational mechanisms.

In line with previous findings that the βAR-cAMP-Epac1-PI3K-mTORC2 pathway in BAT activates Akt in response to cold (Albert et al., 2016; Labbe et al., 2016), the phosphorylation levels of Akt at Ser473 reached their peak between 1 and 2 days of cold exposure (Supplementary Figure S1). Although direct assessment of mTORC2-mediated phosphorylation of SGK1 at Ser422 (Pao et al., 2010) was not possible due to rapid SGK1 degradation, the kinetics of SGK1 activation, indicated by increased phosphorylation of NDRG1 at Thr346, paralleled those of mTORC2-dependent Akt activation, peaking between 1 and 2 days of cold exposure (Supplementary Figure S1). Moreover, cold-dependent activation of Akt and SGK1was accompanied by increased phosphorylation of their downstream substrates, as measured by a phospho-RXXS/T motif-specific antibody (Supplementary Figure S1). While both Akt and SGK1 share similar consensus phosphorylation motifs (RxRxxS/T or RxxS/T), SGK1 is shown to be the primary kinase responsible for phosphorylating NDRG1 at Thr346 (Sommer et al., 2013; Mason et al., 2021). In support of this notion, both pharmacological and genetic inhibition of SGK1 in ISO-stimulated brown adipocytes led to a marked reduction in NDRG1 phosphorylation at Thr346. While global *Sgk1* knockout mice do not display significant phenotypic changes under normal conditions, they have been shown to exhibit specific physiological alterations, such as reduced sensitivity to hypoxia-induced pulmonary arterial hypertension (Xi et al., 2019) and impaired sodium retention on a low-salt diet (Wulff et al., 2002). In contrast to the reduced mitochondrial respiration observed in *Sgk1* ^−/−^ brown adipocytes, BAT-specific *Sgk1* knockout mice (*Sgk1* ^BKO^) maintained cold tolerance during exposure to 4°C (data not shown), suggesting the presence of compensatory mechanisms that may be activated in the absence of SGK1 activity. Future studies will be necessary to identify the compensatory mechanisms responsible for the cold tolerance in *Sgk1* ^BKO^ mice. In addition, the full spectrum of SGK1 substrates in brown adipocyte remains to be defined in order to comprehensively understand the broader cellular functions of SGK1 during cold activation.

In summary, our findings identify SGK1 as a novel signaling component that regulates GOT1 protein stability in brown adipocytes during cold exposure. Distinct layers of regulation – PKA-medicated control of *Got1* transcription and SGK1-mediated stabilization of GOT1 protein – work in concert to maintain GOT1 protein levels during thermogenic activation. These findings not only expand our understanding of SGK1’s functional repertoire but also provide new insights into the dynamic regulation of metabolic enzymes in adapting to cold-induced metabolic demands.

## Data Availability

The original contributions presented in the study are included in the article/Supplementary Material, further inquiries can be directed to the corresponding author.
