# Supplementary material for: The β-adrenergic receptor-SGK1 signaling pathway in brown adipocytes protects GOT1 from proteasomal degradation
Source: Front Cell Dev Biol. 2025 Jul 16;13:1637770. doi: 10.3389/fcell.2025.1637770 (PMC12307365; doi:10.3389/fcell.2025.1637770)
Supplement: Supplementary file 1 [file DataSheet1.pdf]

The β-adrenergic receptor-SGK1 signaling pathway in brown adipocytes protects GOT1 from proteasomal degradation

Chul-Hong Park[1], Minsung Park[1], J. Jason Collier[2], Ji Suk Chang[1]*

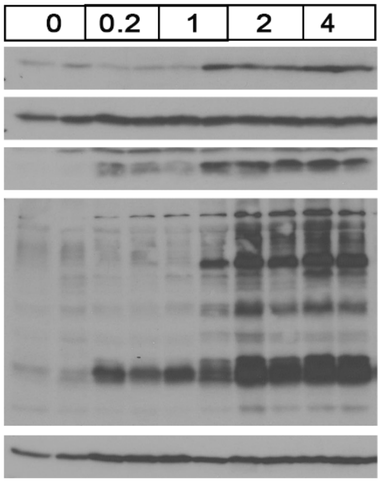

Supplementary Figure S1. Effect of cold exposure on β-adrenergic receptor downstream signaling pathways in BAT. Western blot analysis of brown adipose tissue (BAT) harvested from C57BL/6J mice that were exposed to 4°C for the indicated durations.
